# Information sharing in interteam responses to disaster

**DOI:** 10.1111/joop.12217

**Published:** 2018-04-01

**Authors:** Sara Waring, Laurence Alison, Grace Carter, Chloe Barrett‐Pink, Michael Humann, Lauren Swan, Tomas Zilinsky

**Affiliations:** ^1^ Critical and Major Incident Psychology Research Group Psychology Department University of Liverpool UK

**Keywords:** multiteam system, information sharing, knowledge boundaries, representational gaps, emergency response

## Abstract

Research demonstrates that information sharing is facilitated by familiarity, and having a common understanding of problems, use of lexicon, and semantic meaning. These factors can be difficult to develop within extreme environments such as disasters as members of the multi‐agency system that responds often have limited experience of working together. Public inquiries repeatedly highlight the impact of information sharing difficulties on public safety, but limited academic research has focused on identifying concrete behaviours that facilitate interteam information sharing within such environments. This paper presents a case study of a national disaster response exercise involving 1,000 emergency responders. Data consist of structured observations, recordings of interteam meetings, and interviews with emergency responders. Results of mixed‐method analysis indicate that interteam information sharing is delayed by limited situation awareness and poor articulation. Conversely, adopting behaviours that promote common frames for understanding interteam capabilities and information requirements improves information sharing and potentially reduces cognitive effort required to process information. Findings contribute to interteam communication theory by highlighting that in complex, time‐constrained environments, having a shared understanding of responsibilities and information requirement is important for minimizing redundant deliberation and improving relevance and speed.

**Practitioner points:**

- Facilitating the exchange and interpretation of relevant information is important for improving situation assessment, decision‐making, and the implementation of appropriate actions for addressing risks.
- Interteam information sharing can be particularly challenging when teams are comprised of members from across different organizations with different language and cultures that must form *ad hoc* to rapidly respond to problems in extreme environments.
- Adopting communication strategies that develop common frames‐of‐reference can facilitate information sharing and interteam responses to disasters.

## Background

Naturalistic decision‐making (NDM) seeks to describe how decisions are made in dynamic real‐world contexts characterized by time pressure, risk, uncertainty, and lack of excessive or incomplete information (Klein, 2008; Lipshitz, Klein, Orasanu, & Salas, 2001). Findings from this domain highlight the importance of having access to relevant information for developing an accurate understanding of what is happening and how this might progress (referred to as situation awareness or SA; Endsley, 1995, 2000, 2015) so that decisions and actions taken are appropriate to the situation (Rankin, Dahlbäck, & Lundbery, 2013). Failure to share or pay attention to relevant information can lead to increased uncertainty and delayed decision‐making (Alison *et al*., 2015), resulting in devastating consequences, as disasters such as the Clapham Rail Crash and Cumbrian Shootings demonstrate (Pollock, 2013). Understanding what facilitates information sharing is therefore vital to improving SA and decision‐making in ‘extreme’ environments, including disasters (DeChurch *et al*., 2011), military operations (DeCostanza, DiRosa, Jiménez‐Rodríguez, & Cianciolo, 2014), and medical emergencies (Mathieu, Marks, & Zaccaro, 2001).

However, the complexity of these contexts and the multiteam systems (MTSs) that form to respond can make it difficult to develop facilitators traditionally associated with effective information sharing, such as familiarity (Ren & Argote, 2011), trust (Jarvenpaa & Keating, 2011), and shared appreciation of ‘who knows what’ (Heavey & Simsek, 2015; Wegner, Guiliano, & Hertel, 1985). Differences in responsibilities, goals, and expertise can create barriers for knowing what information to share with whom, when, and how to interpret information. As advocated by NDM researchers (Burke, Salas, Estep, & Pierce, 2007), it is therefore important to study teams ‘in the wild’ to develop interventions of relevance to improving performance within these contexts.

Drawing on data from a UK Home Office funded disaster exercise, we seek to build upon theories of team communication by identifying concrete behaviours that improve interteam information sharing in extreme environments. Such research poses implications for translating theory into practice and demonstrating the types of activities that aid with balancing shared (Salmon, Stanton, Walker, & Jenkins, 2009) and distributed (Stanton *et al*., 2006) SA across complex networks.

### Disaster context: multiteam systems and situation awareness

Disasters are events that threaten large‐scale damage to human welfare and security, requiring a multi‐agency response (London Emergency Services Liaison Panel [LESLP], 2015). Similar to many countries, the United Kingdom manages these incidents through a three‐tiered hierarchical command structure, with decisions being fed from Strategic (responsible for setting overall objectives) to Tactical (setting parameters and level of autonomy for Operational to work to) and Operational (managing the incident ground) Commanders (LESLP, 2015). This structure is characterized by: (1) high skill differentiation between teams; (2) high authority differentiation with figures from each agency across each layer of command responsible for making key decisions; and (3) low stability as the MTS (team of teams; Marks, DeChurch, Mathieu, Panzer, & Alonso, 2005) forms *ad hoc* in response to an incident and disbands immediately afterwards (for a taxonomy of teams see Hollenbeck, Beersma, & Schouten, 2012).

As with other MTSs, the agencies involved in managing disasters share overarching superordinate goals at the multiteam level (save lives, reduce risks), but unique subgoals (LePine, Piccolo, Jackson, Mathieu, & Saul, 2008; Marks *et al*., 2005) at team and member levels. For example, during a disaster police may need to collect evidence and conduct investigations, whilst fire and ambulance focus on extracting and treating casualties. Common superordinate goals are insufficient for improving performance (Power & Alison, 2016); component teams must also coordinate and prioritize the order in which subgoals are addressed to avoid conflicting actions (Mathieu *et al*., 2001). Exchanging, integrating, and interpreting information is therefore important for informing SA at individual (Endsley, 2000), team (Endsley & Jones, 1997; Wright, Taekman, & Endsley, 2004), and multiteam levels to ensure that subgoals between teams and agencies are compatible and directed towards achieving superordinate goals (Davison, Hollenbeck, Barnes, Sleesman, & Ilgen, 2012). However, the extent to which teams across the MTS need to have a shared situation awareness (SSA) in order to promote effective coordination of subgoals requires further focus.

At the individual level, SA is important for ensuring that decisions made are appropriate to the situation and are implemented in a timely manner (Alison *et al*., 2015). Effective teamwork requires that all members have the SA needed to address their responsibilities, referred to as team situation awareness (TSA; Endsley & Jones, 1997). For TSA to develop, knowledge of team roles, capabilities, and interpersonal relationships are needed (Berggren, Johansson, Baroutsi, Turcotte, & Tremblay, 2014), along with an element of SSA across members to promote coordination (Cooke *et al*., 2003). However, seeking to develop complete SSA across the MTS and to share all information with everyone would become overwhelming, resulting in attention being misdirected away from important aspects of tasks (Stanton *et al*., 2006).

Accordingly, researchers studying cognition within complex networks highlight the value of taking a systems approach that sees cognitive processes as being dispersed across members rather than solely residing within individuals (Stanton *et al*., 2006). SA is viewed as being distributed (DSA), connecting members to a task on a ‘moment‐by‐moment’ basis (Stanton, 2016). Similar to the concept of MTSs in which teams have different subgoals that need to be coordinated to achieve a superordinate goal (Davison *et al*., 2012), DSA implies different, yet compatible, goals and information requirements that may sometimes overlap for particular tasks (Nazir, Sorensen, Overgård, & Manca, 2014; Saner, Bolstad, Gonzalez, & Cuevas, 2009). Thus, effective information sharing does not mean that all information should be exchanged with everyone, but rather that each member has access to information of relevance to the function they serve when they need it (Stanton, 2016; Stanton *et al*., 2006).

In this respect, DSA is not only beneficial for understanding the importance of being selective in sharing information, but also provides criteria for judging the quality of information shared based on relevance to recipients’ functions. However, DSA provides less focus on explaining how this can be achieved. Identifying behaviours that promote ability to share and make sense of relevant information within complex networks such as MTSs is important for improving practice.

### Information sharing

Macrocognitive research into complex cognitive functions in real‐world contexts identifies a range of barriers that can prevent relevant information from getting to where it is needed (Schraagen & Van de Ven, 2011). For example, disasters tend to be managed using a hierarchical, centrally controlled command structure (Schraagen, Huis in ‘t Veld, & De Koning, 2010). This can create barriers for sharing information because the priorities that need to be rapidly addressed often lie at a lower level to where information is being funnelled for commands to be issued (Scholtens, 2008). As different command levels are often geographically dispersed and unable to communicate face to face, the amount of information shared becomes further reduced (Martin & Bal, 2006; Zika‐Viktorsson, Sundström, & Engwall, 2006). Consequently, decision‐makers may have limited access to the information they need.

Conversely, there may be times when too much information is available, placing strain on decision‐makers already operating under cognitive constraints due to environmental complexities (Klein, 2008; Lipshitz *et al*., 2001). According to Data/Frame Theory, adopting a ‘frame’ or internal perspective to make sense of an event can reduce cognitive effort by allowing people to be selective over what information they attend to and how it is interpreted (Klein, Moon, & Hoffman, 2006a, 2006b). This is a two‐way process as data can also influence the frame applied (Baber, Attfield, Conway, Rooney, & Kodagoda, 2016), making it important for decision‐makers to have access to relevant data to test the suitability of their frame. In this respect, Data/Frame Theory provides an important contribution by identifying cognitive mechanisms that influence how people receive, select, and interpret information. However, the theory is limited by the lack of focus directed towards identifying behaviours that can improve access to relevant information, and the influence of the sender on the recipient's ability to recognize information as relevant.

One concept that may shed light on the role of the sender is representational gaps (Kozlowski & Ilgen, 2006). Similar to Data/Frame Theory, representational gaps research posits that how problems are conceptualized affects what information is viewed as being relevant to attend to and share, and how it is interpreted (Bechky, 2003; Mendonça, Jefferson, & Harrald, 2007). However, this concept also highlights that differences in expertise and practices across team members can lead to differences in how problems are conceptualized (Cronin & Weingart, 2007; Weingart, Cronin, Houser, Cagan, & Vogel, 2005). This can result in failures to share information of relevance to others (Sandhåland, Oltedal, Hystad, & Eid, 2015), as well as to implementing actions that work against one another (Cronin, Bezrukova, Weingart, & Tinsley, 2011; Firth, Hollenbeck, Miles, Ilgen, & Barnes, 2015). Findings pose implications for Data/Frame Theory and DSA by indicating that a potential barrier to the availability of relevant information is the extent to which the sender and recipient share a common understanding of the problem.

Evidence also highlights that differences in roles and expertise can lead to disparities in communication between sender and recipient, affecting whether messages can be interpreted. Differences in cultures, command structures, and procedures of agencies involved in disaster response can lead to knowledge boundaries (Kotlarsky, van den Hooff, & Houtman, 2015), including differences in specialist terminologies or using different words to talk about the same object (syntactic boundaries; Bechky, 2003), and attaching different meanings to the same words (semantic boundaries; Boland & Tenkasi, 1995). If members are unable to understand messages, they are unlikely to view them as being relevant to their area of practice (pragmatic boundaries), leading to such information being ignored (Jarvenpaa & Keating, 2011). Knowledge boundaries research poses implications for Data/Frame Theory by highlighting that how information is communicated affects whether the recipient can correctly recognize its relevance.

Further team‐based research identifies that concepts such as familiarity and trust (Jarvenpaa & Keating, 2011; Ren & Argote, 2011) are important for improving the relevance of information sharing. However, these can be difficult to develop during acute phases of a disaster as agencies usually work independently of one another on a day‐to‐day basis (Shuffler, Jiménez‐Rodríguez, & Kramer, 2015), membership is fluid due to changes in working shifts (Allison & Shuffler, 2014), and expertise may need to be combined in new ways to address unique challenges (Goodwin, Essens, & Smith, 2012; Luvison & Marks, 2012).

Thus, many organizations have sought to invest in preparation activities (Maynard, Mathieu, Rapp, & Gilson, 2012) that can be beneficial for improving performance during acute phases, such as gaining work experience across a range of relevant functional domains (Cuijpers, Uitdewilligen, & Guenter, 2016; de Vries, Walter, van der Vegt, & Essens, 2014). Evidence highlights that these activities lead to quicker exchange of relevant information as members become better able to identify what to share (Schraagen *et al*., 2010) and where knowledge resides across the team (in line with transactive memory systems; Wegner, 1987; Wegner *et al*., 1985). It may be that such activities help to reduce representational gaps by promoting shared understanding of one another's responsibilities and the problem. From Data/Frame Theory and knowledge boundaries perspectives, such activities may also be beneficial for developing common frames‐of‐reference for using and interpreting words (Cronin *et al*., 2011; Rentsch & Staniewicz, 2012). These common frames can reduce idiosyncratic perceptions (Dierdorff, Surface, & Brown, 2010), improve accuracy, consistency of judgements (Schleicher & Day, 1998), interteam communication and coordination (Firth *et al*., 2015), as well as freeing up cognitive capacity to focus on other aspects of tasks such as ensuring actions across teams are aligned to shared superordinate goals (Schmidt & DeShon, 2007).

Emergency responders also invest in joint activities such as large‐scale training exercises to familiarize themselves with one another's procedures. However, such activities often rely on voluntary actions (Scholtens, 2008) and are costly to implement, limiting the frequency of their occurrence. In the United Kingdom, agencies involved in disaster response are split into ‘Category 1’ (emergency services, health bodies, and local authorities) who serve a leading role, and ‘Category 2’ (including utility companies and local businesses) who provide support when disasters affect their sector (UK Civil Contingencies Act, 2004). Category 2 agencies tend to be less regularly involved in disaster response and preparation activities, which may limit familiarity, causing disparities in ‘frames’ adopted to interpret the situation. Each incident is also unique, which may raise novel issues regarding roles and responsibilities that previous preparatory activities have not addressed. It is therefore important to consider whether there are behaviours that could help agencies to develop shared understanding of roles, responsibilities, and requirements within the acute phase of an incident in order to better communicate information that will be accurately recognized as relevant.

### Current research

Existing research highlights the importance of sharing relevant information in a manner that can be easily understood if it is to be effectively used to test frames, develop SA, and coordinate subgoals across a MTS. However, theories such as DSA and Data/Frame are limited because they do not focus on the role of the sender and their impact on the ability of the recipient to utilize information. Nor do they identify what concrete behaviours improve ability to share and attend to information within extreme environments. This is an important step for translating theories into practice, developing interventions that train specific skill sets that can be tested.

Accordingly, the current study seeks to address the following question: ‘what behaviours improve and hinder the exchange of interteam information sharing in extreme environments where teams have limited experience of working with one another?’ We take a field‐based approach in order to collect rich, in‐depth data during a Home Office funded national disaster exercise designed to replicate the complexity of a real incident. This approach allows us to focus on behaviours in context (Alison *et al*., 2013), as advocated by the NDM domain (Burke *et al*., 2007).

## Method

### Participants and scenario

Data were collected using naturalistic observation, a method for accessing rich data to develop in‐depth understanding of human behaviour *in situ* (Bashir, Afzal, & Azeem, 2008), allowing interteam communication practices to be observed within an extreme environment. This method is frequently used within NDM research to explore how human cognition adapts to complexity, and is beneficial for developing evidence bases to inform professional practice and training (Gore, Flin, Stanton, & Wong, 2015). Naturalistic observations were made during a 9‐hr exercise that involved 1,000 practitioners from across Category 1 (Police, Fire and Rescue, Ambulance, National Health Service England, Local Council) and Category 2 agencies (Environment Agency, gas, electricity and water companies), Royal Air Force and Government. Additionally, 175 members of the public and actors played the role of casualties, and media agencies were present to generate further realism. To preserve anonymity and prevent exercise disruption, demographics were not recorded.

Overall, the exercise comprised two sites. The first was a physical construction of a train that had derailed and collided with a multistory building (Sector one), several vehicles and power lines (Sector two), and had caused a bus to crash into an adult learning centre (Sector three). Police, Fire, and Ambulance Operational Commanders were based across this incident ground. The second site consisted of a Command Centre approximately five miles away from the incident ground. All Strategic and Tactical Commanders were based here, except for the Fire Service Tactical Commander who was based on the incident ground. A Fire Service Tactical liaison was present in the Command Centre to relay messages and information, as is the usual structure in the United Kingdom.

### Data collection

This study predominantly focuses on information sharing at Strategic and Tactical levels because these levels are responsible for making complex decisions regarding superordinate goals, priorities, and resource allocation. The data have been drawn from across three sources (meeting transcripts, observations, and interviews) in order to increase the rigour and validity of findings through data triangulation (Gelo, Braakmann, & Benetka, 2008).

#### Meeting transcripts

The primary source of data consists of transcriptions of five Tactical and four Strategic meetings that took place across the 9‐hr period, providing an accurate and in‐depth account of communication practices (what was said, by whom and when). These meetings provided a platform for agencies to exchange information in order to understand the situation and risks involved, to identify strategies, assign actions, and consider the shared superordinate goals of saving life and reducing harm. The frequency and regularity of such meetings is not set by protocols, but is dependent on the dynamic nature of each incident.

In total, 15 practitioners from across 12 agencies were present in Strategic and Tactical meetings. Information sharing between agencies at Strategic and Tactical levels took place via verbal communication within these meetings. Information was also fed to these Commanders by a larger network of practitioners located within the Command Centre and on the incident ground using radio communication devices. Each emergency service had a radio channel that was used to share information within the agency across the command structure. Practitioners at Strategic and Tactical levels chose whether to share this information with other agencies verbally.

The average meeting length was 26 min for Tactical and 32 min for Strategic (see Table 1). The first Strategic meeting was particularly long due to Commanders delaying discussions to wait for updates from the first Tactical meeting that had continued beyond the allotted timeslot. As Table 1 shows, this pattern in delayed information sharing across hierarchical levels continued throughout the incident due to overlaps in meetings and media briefings. The impact of this will be discussed in the Results section.

**Table 1:** Tactical and Strategic meeting and media briefing lengths

Tactical meeting length	Strategic meeting length	Overlaps
1st: 37 min (11:39 a.m.–12:16 p.m.)	1st: 47 min (11:55 p.m.–12:42 p.m.)	21 min
2nd: 36 min (13:26 p.m.–14:02 p.m.)	Media briefing 1: 15 min (13:32 p.m.–13:47 p.m.)	15 min
	2nd: 23 min (14:05 p.m.–14:28 p.m.)	
3rd: 19 min (15:02 p.m.–5:21 p.m.)		
4th, part 1: 2 min (15:57 p.m.–15:59 p.m.)		
4th, part 2: 14 min (17:03 p.m.–17:17 p.m.)	3rd: 43 min (16:35 p.m.–17:18 p.m.)	14 min
	Media briefing 2: 19 min (17:37 p.m.–17:56 p.m.)	
5th: 16 min (18:34 p.m.–18:50 p.m.)		
	4th: 17 min (19:34 p.m.–19:51 p.m.)	

#### Observer ratings

Eight subject‐matter experts (SME) observed Strategic and Tactical meetings to assess the quality of information sharing and collaboration between agencies. Four of these observers were academics specialized in decision‐making and communication research, and four were emergency service practitioners with between eight and 37 years of experience (average experience = 17 years). A combination of academic and practitioner SMEs were teamed up across locations to provide a more robust evaluation of performance.

All observers used a standardized observation‐coding sheet in order to provide consistency in the behaviours assessed. Observations were coded in terms of: (1) communication (the extent to which agencies communicated clearly and concisely with one another without using jargon‐ or agency‐specific acronyms); and (2) SSA (the extent to which agencies exchanged information to develop a shared understanding of the incident and how it may progress). Behaviours were scored on a scale of 0 (completely absent) to 2 (consistently present throughout the meeting). Sheets also provided space for general observational notes to explain performance ratings given. Interclass correlations were conducted to measure inter‐rater reliability between the four Strategic (communication: *R* = .73, *p* < .05; SSA: *R* = .71, *p* < .05) and four Tactical observers (communication: *R* = .93, *p* < .001; SSA: *R* = .80, *p* < .05), which indicated a substantial‐outstanding level of agreement. Due to the small number of observations conducted (7 per SME), caution is recommended in interpreting the reliability of this analysis.

In order to allow distinctions in the quality of communication to be identified over time, scores were averaged across three equal time points (10 a.m.–1 p.m.; 1 p.m.–4 p.m.; and 4 p.m.–7 p.m.) and converted into percentages (e.g., 100% = all observers noted a behaviour as being consistently present throughout all of their observations within a 3‐hr time period; 0% = behaviour was consistently absent). This was achieved by adding all observer scores at each time point for Strategic and Tactical levels and dividing by the maximum score achievable based on the number of observations made.

#### Interviews

Semi‐structured interviews were conducted with three Strategic and four Tactical Commanders from across emergency services (mean interview length = 18 min; minimum = 12 min; maximum = 30 min) immediately post‐exercise to capture initial reflections of communication processes during the incident. Questions were developed based on the researchers’ previous experience of observing large‐scale disaster exercises and conducting post‐incident debriefs with emergency services after real disasters. Interviews were semi‐structured, the order in which questions were asked could be altered and additional questions asked depending on interviewee responses.

Overall, interviews initially consisted of questions designed to encourage Commanders to begin reflecting on the incident, such as: ‘What was your role?’; ‘What were you initially faced with?’; and ‘What was the most challenging aspect?’. They were then asked questions relating to communication and information sharing practices, such as: ‘What strategies were used to communicate within your own agency/with other agencies?’; ‘How did you personally seek to ensure effective communication within your agency/between agencies?’; and ‘What do you think were the most important factors that enabled/hindered emergency services from working well together and why?’. These interview transcripts serve as an additional source of qualitative data to contrast with meeting transcripts that are based on the personal reflections of those who were responsible for managing the incident.

### Analysis

Observer ratings are presented in the form of descriptive statistics and are used as supporting data to demonstrate changes in performance over time, based on the assessments of academic and practitioner SMEs. Transcriptions of meetings and interviews were qualitatively analysed using thematic analysis, a form of qualitative analysis that allows common themes to be identified based on content in order to derive meaning (Hsieh & Shannon, 2005). Frequencies are also provided for each theme, in line with a content‐analytical approach (Jones, Coviello, & Tang, 2011), to demonstrate patterns in information sharing practices across meetings over time.

To avoid missing key content within the rich dataset, an inductive data‐driven approach was adopted rather than having pre‐defined themes (Frith & Gleeson, 2004). The first stage of analysis consisted of transcribing audio recordings of meetings and interviews. Video footage was used to support the identification of speakers within meetings. A total of 18,103 words were spoken across Strategic meetings (average per meeting = 4,526), 38,710 across Tactical meetings (average = 7,742), and 18,467 across interviews (average per interview = 2,638). Transcripts were read several times to identify the subset of data relevant to understanding what practices facilitated and hindered information sharing, resulting in 32% of the meeting (17,973) and 13% of the interview data set (2,407) being utilized for subsequent analysis. To improve reliability, a second rater read a random selection of 30% of the larger data set and selected content relevant to the research question. Results of a two‐way mixed‐model intraclass correlation (McGraw & Wong, 1996) found an excellent level of agreement (*R *=* *.91, *p *<* *.001).

In line with guidance provided by Braun and Clarke (2006), a detailed step‐by‐step analysis was conducted on this subset of data. Data were read carefully to identify emerging categories based on similarities and differences in underlying meaning. Data units and categories were then organized into broader themes based on commonalities in content, creating larger data segments (McLeod, 2001). These were compared and contrasted to establish theme boundaries so that differences in the various ways information sharing was either facilitated or hindered could be refined. This process allowed data to be grouped into meaningful concepts that could be discussed in relation to theories whilst still maintaining the language practitioners used (Braun & Clarke, 2006).

To minimize subjectivity, inter‐rater reliability was conducted on 30% of this subset of data with results of a two‐way mixed‐model intraclass correlation showing an excellent level of agreement (*R *=* *.83, *p *<* *.001). Differences were discussed between the two raters, which resulted in absolute agreement.

## Results

In order to explore changes in information sharing and to identify factors that facilitated and hindered these processes, results are presented as follows:

1. SME ratings of communication and SSA at Strategic and Tactical levels across three time points, providing a broad overview of performance changes;
2. Thematic analysis of meetings and post‐incident interview transcripts to provide context and examples of how factors facilitated and hindered interteam information sharing. Frequency of themes across Strategic and Tactical meetings is provided to highlight changes over time.

### Overview of changes in performance

Table 2 provides an overview of SMEs assessments of team performance across the 9‐hr period.

**Table 2:** Performance ratings in Strategic (SCG) and Tactical Command Group (TCG) meetings

	SCG meeting			TCG meeting		
	10 a.m.–1 p.m.	1 p.m.–4 p.m.	4 p.m.–7 p.m.	10 a.m.–1 p.m.	1 p.m.–4 p.m.	4 p.m.–7 p.m.
Communication	90%	75%	79%	68%	44%	100%
Situation awareness	86%	50%	75%	54%	63%	75%
Number of observations	12	8	8	12	8	8

#### Strategic meetings

According to SME ratings, communication and SA development were effective during the early and later phase of the incident, with agencies exchanging information clearly and concisely, and demonstrating shared appreciation of the situation and risks involved (‘Good, clear exchange of information from all agencies. Key lines agreed between three blue lights [emergency services]. Allocation of response to most appropriate persons’ and ‘Especially clear picture of scene and sectors’, SME observers). According to SMEs, prior to most meetings Commanders from Category 1 agencies ‘held smaller informal pre‐briefings in order to exchange information and plan strategies for coordinating activities in the larger meetings’. However, this did not occur before the third meeting, corresponding with SME ratings of poorer communication and SSA. For example, a second incident (helicopter crash) occurred prior to the third meeting, but discussion of this new event was limited, preventing agencies from updating their shared understanding of the situation and how this impacted goals and resources (‘Not clear on new incident. No new review of objectives’, SME observer).

#### Tactical meetings

Effectiveness of communication and SSA improved over time. Initially, the information shared was very limited and lacked detail, creating difficulties for agencies to develop a shared understanding of the situation (‘Scene situation report being collectively discussed but not definitively confirmed amongst Police and Fire colleagues. A collectively agreed and commonly understood situational assessment was not reached’, SME observer). Subsequent improvements in communication and SSA coincided with Commanders from Category 1 agencies permanently collocating outside of formal meetings in order to speed up the exchange of information.

### Thematic analysis

Results of thematic analysis indicate that ability to share and utilize information in a timely manner was hindered by two factors: articulation of information (38.01% of word count) and SSA (11.17%). In line with the concept of knowledge boundaries, the theme of ‘articulation of information’ refers to the ability to communicate relevant information in a manner that can be easily understood by others, such as avoiding use of acronyms, agency‐specific terminology, or irrelevant information. In line with the NDM definition of ‘SSA’, this theme refers to the ability of agencies to form an accurate shared understanding of what is happening on the incident ground and how this might progress. Ability to effectively share information was facilitated by two factors: rationale (40.25%), and roles and procedures (12.35%), both of which relate to Data/Frame Theory and frame‐of‐reference, as will be discussed. The theme of ‘rationale’ refers to members providing a reason for why information was requested or provided. The theme of ‘roles and procedures’ refers to agencies providing clarification regarding their own roles and procedures in relation to the incident or making reference to those of another agency.

Similar to patterns in SME ratings, barriers were predominantly higher in the first and second Tactical meetings (see Table 3) and the third Strategic meeting (see Table 4). This coincided with longer meeting durations (see Table 1). Facilitators were also predominantly higher in these same meetings, suggesting an inability to discriminate between effective and ineffective information sharing strategies. As will be further discussed, problems related to agencies sharing too much information or redundantly repeating discussions rather than ensuring that the information shared was concise and relevant to recipients (as would be advocated by DSA). This resulted in meetings lasting longer than scheduled and prevented information from being exchanged across hierarchical levels. Direct quotes are provided (see Table 5) to support themes, but identifiers have been removed in order to preserve anonymity, most notably practitioner names [name], and locations [location]. The symbol ‘xxx’ refers to content that was inaudible or indecipherable.

**Table 3:** Percentage of facilitators and barriers to information sharing across Tactical meetings

	1st	2nd	3rd	4th	5th	Total
Facilitator						
Rationale	6.62	2.09	4.85	4.41	2.35	20.32
Roles and procedures	2.49	1.61		2.14		6.24
Total	9.11	3.70	4.85	6.55	2.35	26.56
Barrier						
Articulation of information	1.87	4.99	1.81	2.62	5.05	16.34
Inaccurate situation awareness	2.77	5.21				7.98
Total	4.64	10.20	1.81	2.62	5.05	24.32

**Table 4:** Percentage of facilitators and barriers to information sharing across Strategic meetings

	1st	2nd	3rd	4th	Total
Facilitator					
Rationale	2.79	5.04	6.49	5.54	19.36
Roles and procedures	2.35	0.39	2.51	0.76	6.01
Total	5.14	5.43	9.00	6.30	25.87
Barrier					
Articulation of information	3.58	2.02	14.03	0.05	19.68
Inaccurate situation awareness	1.44	1.44		0.27	3.15
Total	5.02	3.46	14.03	0.32	22.83

**Table 5:** Quotes to support themes

Example	Source	Quotes	Explanation
1 Inaccurate situation awareness (SA)	Tactical meeting 1	British Transport Police 1: *I'd get initially the same thing that it was a bus, a car not a bus* British Transport Police 2: *It's one vehicle that's been collided with involving a train. There is no, it was described as a multiple RTC [road traffic collision] before, it's not, several vehicles now* British Transport Police 1: *There are several vehicles involved that exact circumstances how that's come about I'm not aware* Police: *Okay [name] do you know xxxx or was it unattended?* Ambulance: *My [name] said the RTC involved 15 vehicles including a bus which had turned over but it didn't say that it was a school bus, it just said it was a bus* British Transport Police 2: *I mean that's that's something that we got to get a grip of really, really quickly as to is it just a car on a crossing or it is something different and somebody left the scene. Again [name] and I as SIO [senior investigating officer] and SIM [senior incident manager] will be looking at that to determine obviously is there any potential culpability*	Lack of initial information available resulted in difficulties for Tactical to develop an understanding of risks present on the incident ground, including vehicles and hazardous materials, along with how sectors were connected. Much of this information was still unavailable to Tactical during the second meeting, despite Strategic already having access to these details, hindering the ability of Tactical to develop accurate SA. This lack of SA development prevented agencies from identifying what information they would need to update their assessment of risk, resulting in a key agency, the Gas Company, not being invited to earlier Tactical meetings. Tactical were therefore delayed in accessing this important risk information until the third meeting
2 Inaccurate SA	Tactical meeting 2	Police: *Sector 2 is just behind the xxx house this is a train carriage, which has overturned. In terms of that [name]?* Fire: *We have a derailed carriage there umm several vehicles also involved umm 18 casualties that have been removed from that site, 4 casualties are still on scene and being rescued and at this moment in time 15 unconfirmed erm fatalities that is being confirmed at this moment in time and we also have a vehicle with 1 female is and she is being rescued as we speak* Environment Agency: *Again sorry can I just check the vehicles that they're just cars is just to check are there are HGV's [heavy goods vehicles] or carrying certain chemicals* Police 2: *I'll get that information for you* Police: *Sector 3 is xxx café there we've got a railway carriage gone into a building that's roughly where the level crossing was that area* Fire: *Okay umm this we've got a train and building we've got 40 casualties on the carriage and 7 confirmed fatalities. We had it confirmed by BTP that the electricity supplies have been isolated. At the moment in time we've got two USAR teams working on the collapsed structured and within the erm carriages to ensure that there are no other casualties in the buildings or the carriage* Police: *And sector 4 is roughly this area here as you look at the map to the right of Jake's café which was the tower 2 casualties have been moved from the area by helicopter there's a HGV and a car crashed in and around that area is this it* Fire: *Yeah at this moment there is no fire service activity going on in sector 4* Police: *Well the number of fatalities in that area as well?* British Transport Police: *Sorry I couldn't see the map. Are these all connected? I know we're dealing with positively connected link all these incidents are linked?* Fire: *Yes this is all the result of the one incident*	
3 Inaccurate SA	Tactical meeting 3	Gas: *The medium pressure network has been closed down so we wouldn't be looking at further evacuation of the 500 meters at the moment. The low pressure is still connected. We're paying particular attention round the flats to verify if the flats did have gas in there but I must stress that there still is a danger of explosion within the low‐pressure network. We're taking readings but it's difficult for us to get near the site so we are trying to close sections off rather than going but that will take another hour two hours* Police: *So [name] did you say that there is a danger of explosion?* Gas: *There still is a danger because the low pressure network is still connected we could have pockets of gas in the buildings so it's still a danger and that must* Police: *Right and what would your advice be around those particular buildings at this point?* Gas: *If there's no life no life no bodies or no people in there stay away don't go near until we can isolate the gas* Police: *And have you absolutely identified where those structures are and which of those buildings are you referring to?* Gas: *Well that's what we are doing now but the problem is getting access to these buildings because it's chaos* Police: *Cause what I've heard so far about it it's that it's our biggest risk so far I've heard during this meeting so as an urgent action I need you to go away and come back and liaise with NAME (*G: *Yeah) to establish exactly where those buildings are*	
4 Inaccurate SA	Interviews	*I err responding initially to the scene, which is…that's what I would do in order to err to develop the best possible situational awareness that I could to make sure that I could get eyes on an err ensure that I was happy with the way that it was structured from a fire and rescue perspective and the tac commander, the tactical objectives were err appropriate and in keeping with what it was*. *Well, the first informative message was err probably sent within about 2 or 3 minutes. Major incident make pumps ten and maybe err requested the search and rescue team. That is [poor], where is your methane message… You haven't said why, nothing. You know you've done nothing there in the way of sizing that incident up*.	Initially, Strategic Commanders had access to more detailed information regarding the incident than Tactical, due to the Fire Strategic Commander attending the incident in person, whereas Tactical were reliant on limited messages provided from the incident ground
5 Articulation of Information	Strategic meeting 2	British Transport Police: *And of course we talked about a casualty clearing station, initially that's where they're going to. Nothing more to add to that at the current time*. Police: *Ok when you say in terms of casualty reception, what has something been set up in terms of?* British Transport Police: *Which is I think one of our colleagues just updated to that effect* NHS England: *I mentioned about a casualty clearing station but not in terms of survivors reception* 10 minutes later… Local Council: *We are providing all the support we can to our partner agencies. We have set up a survivor reception center and a family and friends center (P: right that's been set up has it? Great) yes (P: and where that is?) [location name]*	Differences in the use of terminology created issues for agencies to understand when the information they had was of relevance to discussions, resulting in delays to sharing information and repetitive discussions
6 Articulation of Information	Tactical meeting 2	Police: *Ok since an xxx an xxx to be drafted and agreed have an update of [name] and lieutenant [name] been completed (*Police 2: *they've done xxx ok thank you) and command in operations and resilience going forward to be looked at by all partners and that's on‐going in relations to xxx (Police 2: ok you can address that in the xxx ok thank you) and an update by the xxx coordination group to be drafted which is done (*Police 2: *yeah) and silver commander to link xxx in relation to outer cordon reliance issues which has been tasked in to our silver erm attendance at gold to be all level representation to be in regular contact with gold so that we are co‐located effective. RVP management and implications to traffic management to [location] road being communicated to our silver commander in term of logistics erm. At the next SCG all gold of mutual aid being requested and where from (*Police 2*: ok) that's starting to look like xxx and multi agencies to xxx an area to keep requests as a matter of priority xxx a policing perspective and the rest to be set up outside the main cordon in sector 4 in a hanger, I don't have a specific location at this time and gold commanders to ensure that strategic objectives are communicated through silver but on a transmission to bronze commanders so strategy turns into the right application (*Police 2*: right) being communicated through*	Failure to be concise and to filter information to ensure that it was of relevance across agencies created difficulties in interpreting messages and identifying relevant cues amongst less relevant
7 Articulation of information	Tactical meeting 5	British Transport Police: *We looked at identifying any significant areas of significance in the investigation around the potential causation. We identified this in the meeting with the RAIB the ORR health and safety etc. My DI investigative bronze and the scene evidence recovery manager and we agreed and agreed protocols around the half barrier at xxx crossing the carriage in situ on the track erm the driver's cab which is the cab on the carriage that's on its side off the track the signal LL1234 the signal box at [location] the tread‐all which is I'm reliably informed is a device that's tripped by the train the train trips the device and that sort of trips across in to operate also the relay room this is the tread‐all talks to the relay room that triggers a crossing erm and these are the key areas that we've looked at. As to where anything that they're working effectively there are no faults and as to whether any significant causation factors those are all things that will take a little bit of time and a specialist from [agency name] will look at those. All of the exhibits processed in a place whereby we make sure the integrity continuity of exhibits and the xxx is in operation in respect of that around the body recovery I might be just slightly out of date now this is this was from the last meeting erm there's only one sector sector 2 which is the cab that the SIM team were really able to get into all the others were held up by through hazards and the fire service were had priority over all of those areas. Erm the sector 2–3 bodies were recovered at the last count leaving 18 bodies still to recover and there are also 3 bodies that have been in situ at the temporary body recovery area when the SIM team arrived there erm the fire service have now made the decision that in in these other sectors they're gonna have to actually dismantle the buildings that would take some considerable time probably a couple of days so it would be a while xxx erm on those those enquiries on‐going HM coroner fully updated*	
8 Articulation of Information	Interviews	*There was almost a surge of information erm in the tactical meetings there was a room full of endless people giving information which everyone sits and listens to but ultimately you spend a long time listening and it wasn't necessarily of any value to me…I need to know my stuff so it was almost as if we needed two meetings*. I know you can go and argue that you all need to have a clear picture of everything that is going on but the reality of environment agencies considerations affecting the ambulance response in that acute phase is nil. Unless its gonna cause us a hazard in which case they need to put their hand up at the beginning and say look there's a real hazard here to x for your staff. I think we were having a spot of bother earlier on during the day because the TCGs were going on too long and so by the time they'd finished all the communication and intelligence and everything we couldn't be late back to our strategic because at times he was going blind erm we weren't getting the information from the front because there was that much going on so therefore then you've got this delay I think the fact that we spent so long in briefings made it difficult to spend time briefing downwards and upwards for me TCGs were overrunning and they were over running so badly that there was no opportunity for him to come out and give me a briefing before going into the SCG so all the information that I'd got was either old, outdated and I couldn't get that information because the tactical coordinating group was going on too long. *I think erm different terminology, erm certainly I got there's a couple of things I'll have to brush up on myself, the acronyms used particularly around the investigation of the DVI [disaster victim identification] personally I would have to get to grips with…I think those are the things that hinder us*.	Delays to information sharing and repetitive discussions resulted in longer meetings, leading to overlaps between Strategic and Tactical meetings and preventing the exchange of information within teams across hierarchical levels
9 Rationale	Strategic meeting 1	Fire: *Based on previous incidents of this nature this is not something that's gonna get resolved quickly from our perspective. You're talking this will run into days and potentially into weeks, not least with the building collapse side of the incident. There are evidence of successful rescues being carried out 14 days after that and it may well take that long to get to err delayer the building to the point where we can say with certainty that we've accounted for everyone so just to make that clear that this is not going to be over quickly will it (P: yeah). Not necessarily going to impact on everyone's resources other than necessarily ours but just to make that point*. Police: *Ok got that, I understand*. Ambulance: *We also got a situation with a number of casualties, there are multi‐trauma cases, there are sever injuries which are likely to exceed the available capacity within the local [city name] area and the wider [county name] area. We are working to try and identify available trauma sites that can be used to potentially take some of the more serious casualties out due to the numbers involved, just to make you aware that we will advise as the day goes on*.	Providing a rationale for information being shared regarding the duration of the incident allowed other agencies to consider the implications of this in terms of their own resource management and to share similar details of how this would impact on managing casualties. Similarly, providing a rationale for why information was needed regarding school attendance enabled quick access to this information as agencies were able to understand what was needed and why
10 Rationale	Tactical meeting 1	British Transport Police: *I would want to know very, very quickly who basically hasn't turned up for school you know. I would wanna know school register who basically hasn't turned up because they may be some of our casualties you know and I would need to get a grip of that quickly*. Police: *Ok can the local [council] take that away?* Local Council: *Yeah, we'll do that*.	
11 Rationale	Interviews	*In all seriousness that was really challenging, even to make people understand. Do you understand what it is I'm asking for?* *We got to the point, mid‐afternoon when the police said to us look it's gonna take us a significant amount of time to be able to answer that question. Now at that point I said right that's fine… This operation will not be resolved in a matter of hours, this is days possibly weeks and you need to understand that and I explained to them what we were gonna do. Because that does then change completely the dynamic. Once the police are telling me I can't tell you, we just don't know, that's fine. That's all I need to know because now we will accept that it has to be dismantled brick by brick if necessary until we get it where we need to get to*.	Although ensuring that other agencies understand the relevance of information requests can be cognitively demanding, agencies recognized the importance of this for accessing information
12 Roles and Procedures	Strategic meeting 3	Fire: *Chair, just in relation to one further matter requiring urgent action I need to make a request to military colleagues [name] military aid to the civil authority CAT A request to the effect that I require is the movement of urban search and rescue teams and assets onto scene ‘cos they are physically unable to get on to err onto the site because of the limitation of the err as caused by the incident so wanted to just flag that up now ‘cos that is an immediate priority for us ok?* Royal Air Force: *Ok, I'll enquire heavily into that because I think it's absolutely right it sounds right*.	Demonstrating a shared understanding of one another's roles and remits allowed quicker coordination, and prevented lengthy discussions on issues that were of less relevance to others within the wider multi‐agency meeting
13 Roles and Procedures	Strategic meeting 3	Fire: *Just on that point chair if I may what we can do is we do have existing protocols with BTP and RAIB [Rail Accident Investigation Bureau] from the USAR [Urban Search and Rescue] capability around firstly body recovery simultaneous with live rescue and also evidence recovery may be worth us now taking. I'll take an action to make sure that we are then enacting that because it will probably allow us to join some of that up a little bit better cos you are clearly being frustrated and doing what you need to do* British Transport Police: *Well there's an understanding that your job takes priority clearly erm but it might just make it slicker if we do that* Fire: *We'll take an action and we'll pick that up outside the meeting*	
14 Roles and Procedures	Interviews	*We've got a shared understanding of each other's resources, principles and priorities and objectives so when we do come together erm… those kind of principles come together to make it an easier relationship, therefore you get more from it and you're better able to manage a scene because you can describe issues which are ongoing and you can have a consensus around decisions* But we've got a pretty good arrangement both through the [Local Resilience Forum], you know we know each partner and there's a lot of mutual respect between the organizations which is very healthy so in terms of my own planning, my own training I've worked with a lot of these guys…So err whilst I don't know all the players by name I'll work with them at some stage and that really helps *It was highlighted on the course about tactical fire they go on the scene or having that tactical in the tactical group which is the same rank as myself, helped an awful lot because we were told that they would send an advisor*	Category 1 agencies noted the value of prior experience of working with one another for facilitation coordination and exchange of information

### Barriers

#### Shared SA

At a Tactical level, limitations in understanding what was happening on the incident ground, why and how this might progress were particularly prevalent during the early phase but reduced as the incident progressed (Table 3). During the first Tactical meeting, members only had access to minimal information about the incident, resulting in difficulties with developing an accurate understanding of the key risks involved such as types of hazardous materials, and how the three sectors linked to one another (Table 5, examples 1 and 2). Much of this information was still unavailable during the second Tactical meeting, despite being discussed during the first Strategic meeting.

Strategic initially had access to more information as a consequence of the Fire Commander attending the incident ground to gain a firsthand account of what was happening. However, Tactical were reliant on formal messages sent via radio from the incident ground, which initially lacked detail (example 4). Overlaps in initial meetings prevented Strategic from sharing more detailed information with Tactical, and such information was not forthcoming from the incident ground during this period, resulting in differences in understanding between hierarchical levels. As a consequence, the ability of Tactical to identify key priorities on the incident ground and to coordinate the activities of Operational teams was affected, leading to delays in emergency responders attending to all three incident sectors (e.g., over an hour before responders were deployed to rescue and treat casualties in sector three).

Not only did lack of information impact on the development of SA, but initial limitations in understanding the situation subsequently made it difficult for Tactical to identify what additional information they would need to enhance their SA, leading to further delays in accessing relevant information. Thus, there appeared to be a reciprocal interaction between SA and accessing relevant information. For example, a representative from the gas company was not requested to attend the first Tactical meeting, despite reports of a potential gas leak on the incident ground being known to Strategic. This resulted in Tactical making decisions without a comprehensive appreciation of risks (e.g., responders deployed to zones at potential risk of gas leaks and explosions). This key information regarding gas risks only became available during the third Tactical meeting (example 3).

#### Articulation of information

There were a number of occasions when the information available to one or more members of the group was not shared at the point in time when it would have been most relevant to do so. This was usually the result of agencies failing to articulate information in a language that others could easily understand due to use of acronyms or words with different semantic meanings. Consequently, agencies were unable to identify when the information they had held relevance to discussions, resulting in them returning to topics already covered to provide delayed information updates (example 5). These repetitions were a key cause of meetings overrunning and overlapping across hierarchical levels.

Messages also sometimes contained an excess of detail, not all of which was relevant to other teams (examples 6 and 7). This was the result of members seeking to provide all of the information their agency possessed rather than matching the information provided to the requirements of the audience and filtering out irrelevant information. Consequently, meeting lengths were further prolonged (example 8). At a Tactical level, discussions became more concise by the third meeting (Table 3), which coincided with Category 1 responders deciding to remain permanently collocated. This smaller group was able to more readily exchange, discuss, and make sense of information and agree common frames or viewpoints so that relevant information could be sourced in a more constructive way during larger meetings. At a Strategic level, the lack of conciseness during the third Strategic meeting also coincided with changes in coordination. Whereas Category 1 responders held smaller briefings to share and integrate information prior to the first, second, and fourth meetings, they were unable to do so in advance of the third meeting, affecting their ability to keep discussions more focused to access relevant information during the larger meeting.

### Facilitators

#### Rationale

Across the course of the incident, there were many occasions when members provided a rationale for why information was being shared, particularly with regard to resources and incident management (example 9). Similarly, there were many occasions when members provided a rationale for why information was needed (example 10). From a Data/Frame Theory perspective, it is possible that these activities helped to clarify the frame that the speaker adopted making it easier for others to interpret what was needed.

In line with this, during post‐incident interviews Commanders noted that getting others to understand why they needed information could be challenging but was important for quickly accessing this so that plans could be formulated to address goals (example 11). For example, Fire needed details of the total number of people known to be located across the incident ground in order to provide a stopping point for investing resources into conducting rescues. Police were responsible for sourcing such information, and once they understood the significance of this for achieving shared goals, they informed Fire that the information could not be sourced to the level of accuracy needed to be confident that all casualties were rescued. Fire was then able to alter their strategy and resource plans accordingly, accepting that the scene would need dismantling to ensure no casualties remained unaccounted for. Thus, the operation would last for a period of days or weeks rather than hours.

#### Roles and procedures

Another facilitator of effective information sharing was providing clarification regarding the roles and procedures of component teams. Not all teams were equally familiar with one another (e.g., Category 1 and 2 responders were less familiar with one another), and so providing this type of information appeared to enable them to develop a shared ‘frame’ regarding capabilities across the MTS for dealing with the specific incident. Accurately demonstrating knowledge of another team's roles was also beneficial for gaining quicker access to relevant information. For example, the Fire Commander's accurate understanding of the roles and remits of the Royal Air Force during the third Strategic meeting allowed quick access to the assistance needed for transporting heavy specialist equipment to the scene using Air Force assets (example 12).

Similarly, in recognizing the roles and remits of British Transport Police, Fire was able to quickly provide support to this agency (example 13). Whilst Fire's subgoal was to rescue casualties, British Transport Police needed to secure evidence to investigate the cause of the rail incident and assess whether it was a criminal matter or accident. Both parties understood one another's subgoals and had a shared understanding of the order in which these needed to be prioritized. Such knowledge also enabled them to quickly identify mutually beneficial strategies. Both parties also understood that the problem was specific to their agencies and arranged to discuss it separately rather than causing disruptions by discussing information of irrelevance to other component teams within the wider meeting.

During post‐incident interviews, Commanders reflected on the importance of being aware of one another's roles, remits, and procedures for accessing the information needed (example 14). In particular, practitioners noted how useful training or previously working together was for gaining such knowledge prior to an incident where possible.

## Discussion

This research aimed to identify examples of concrete behaviours that facilitate and hinder interagency information sharing in extreme environments. Findings highlight that poor SSA, use of agency‐specific terminology, and failure to be concise delay interteam information sharing. Conversely, adopting communication behaviours that create common ‘frames’ for interpreting messages and understanding information needs, such as providing a rationale for why information was requested or provided, and articulating roles, and responsibilities facilitates the sharing of relevant information. Conducting smaller, spontaneous briefings with agencies that have primacy over managing an incident helps agencies to process information in order to promote more concise and targeted focus on sourcing and sharing information during larger meetings, potentially reducing cognitive load. Taken together, findings indicate that both promoting shared frames and adopting a networked structure can improve the relevance and speed of information exchange within dynamic MTS environments.

### Information sharing barriers and facilitators

As NDM research highlights, the complexity of dynamic environments often places decision‐makers under increased cognitive load (Klein, 2008; Lipshitz *et al*., 2001), reducing capacity for processing information (House, Power, & Alison, 2014). DSA highlights the importance of sharing information of relevance to the function of the recipient in order to improve their ability to complete their function without becoming overloaded (Stanton *et al*., 2006). From a Data/Frame Theory perspective (Klein *et al*., 2006a, 2006b), cognitive effort can also be reduced by adopting ‘frames’ that allow people to select what information they attend to. The current study sought to advance DSA and Data/Frame Theory by identifying concrete examples of behaviours that can improve information sharing, making abstract concepts more tangible for practitioners to adopt. Whilst Data/Frame Theory focuses specifically on how individuals selectively attend to information, we also focus on the role of the sender and how their communication behaviours can influence whether the recipient attends to information.

Overall, findings suggest that there are concrete behaviours that both the sender and recipient can adopt to improve access to relevant information. For example, in line with knowledge boundaries research (Kotlarsky *et al*., 2015), findings suggest that information should be communicated by the sender in a way that makes it easier for the receiver to understand and recognize the relevance of. This can be achieved by avoiding specialist terminologies (Bechky, 2003) and adopting common ‘frames’ for interpreting words (Boland & Tenkasi, 1995). In contrast to previous research, findings also demonstrate that differences in using and interpreting terminologies can result in delayed information sharing across the MTS. For example, practitioners being unable to identify the relevance of the information they possessed to discussions taking place due to inconsistencies in words used to describe the same objects and concepts led to repetition as they returned to topics already covered to introduce new information. This caused interagency meetings to overrun, creating disparity in SA and disrupting the ability for command levels to update one another.

It should be noted that, in the context of how these teams usually operate, it is unusual for a member of Strategic Command to attend the incident ground to gain a firsthand overview of the scene. Consequently, it is also unusual that Strategic would have greater access to operational information than Tactical as information would usually be passed from the incident ground to Tactical who would provide a condensed version to Strategic. However, initial inability to share information across command levels had a long‐standing impact on Tactical, delaying their ability to identify what further information would be needed to improve SA (e.g., recognizing the potential for a gas leak and requesting gas company representatives to be present in meetings). Given that disasters are often characterized by a need to complete tasks urgently (Klein, Ziegert, Knight, & Xiao, 2006), restrictions to receiving and making sense of information pose implications for rapidly responding to threats (Rankin *et al*., 2013). The knock‐on effects of barriers to information sharing across command levels on identifying what was happening and how to reduce risk highlight the importance of identifying ways to promote access to better quality, relevant information from the incident ground, particularly during early incident phases.

Findings also reinforce the importance of only sharing information that is relevant to the functions of the other agencies attending interagency meetings and delivering messages concisely. From a DSA perspective, sharing too much information increases the cognitive load of the recipient (Stanton *et al*., 2006). Current findings indicated that failing to concisely communicate messages resulted in agencies disengaging because they felt this information was irrelevant and prolonged interteam meetings unnecessarily. As with failing to communicate using a common, shared language, failing to be concise also caused meetings to overrun, which prevented information from being distributed across the wider network. This suggests that in a time‐constrained MTS, one way in which DSA may be improved is by communicating concisely and using a common language ‘frame’.

Additionally, findings indicate that providing a rationale for why information is being requested or shared can both speed up access to and encourage attention to be paid to relevant information by developing a common frame for interpreting information requirements. This may enable members to understand the relevance of information for coordinating subgoals, potentially encouraging them to pay attention to messages by making them easier to interpret (Jarvenpaa & Keating, 2011). Similarly, providing information about agencies’ roles and remits also appeared to create a common frame for viewing the problem, how each agency was seeking to resolve it and overlaps in subgoals, enabling agencies to better understand one another's capabilities, where coordination is required and what information needs to be shared as a consequence.

Findings also indicate that holding smaller briefings prior to larger meetings can reduce redundant and repetitive deliberation. Schraagen and Van de Ven (2011) have previously highlighted the value of adopting decentralized networks rather than centralized authority structures for improving access to information during disaster management. Within networked structures, information is shared horizontally and vertically throughout the organization so that people are able to access the information needed, even if the source of this information comes from another team, unit, or agency. Within the current disaster exercise, there were examples of Category 1 agencies seeking to adopt aspects of a network structure alongside the hierarchical structure, such as Tactical responders deciding to remain permanently collocated, and Strategic responders conducting pre‐briefings prior to meetings. In line with Schraagen *et al*. (2010), adopting these network strategies enabled these responders to share and make sense of information more rapidly.

Forming these smaller collective groups may also prevent information overload by allowing responders to strike a better balance between managing information sharing and coordinating subgoals. For example, Category 1 responders were able to utilize larger meetings to provide a coordinated overview of the incident and actions underway, and to focus on seeking information from Category 2 agencies to inform planning for particular subgoals. Consequently, meetings were shorter in duration, leading to fewer overlaps and allowing information sharing between command levels. These findings suggest that, in dynamic environments, adopting network structures may be beneficial for promoting a balance between SSA and DSA, allowing agencies to distribute information more efficiently. However, in order for information to be shared in the first instance, substantial efforts still need to be made to manageably obtain and understand information before it can be shared. This requires a balance between investing resources for gathering and sharing information, as obtaining and exchanging information simultaneously is difficult.

### Limitations

A qualitative approach was adopted to contribute to understanding what facilitates interteam information sharing in extreme environments. Qualitative analysis is often criticized on the grounds of subjectivity, which poses implications for data interpretation. To address this, inter‐rater reliability was conducted with a second rater and interview transcripts and SME observations were used as additional sources for data triangulation. However, it would be beneficial for future research to conduct more in‐depth interviews to explore practitioners’ personal reflections of the behaviours that improve information sharing and compatible interpretation. Although this research demonstrates the value of developing shared frames for understanding roles and remits, and information requirements, these strategies may require communicators to expend additional cognitive resources to focus on what information to provide, why and how to communicate this in a way that can be easily processed by others. Conducting in‐depth interviews using methods such as cognitive task analysis may be beneficial for understanding these trade‐offs.

Additionally, as the data were generated during a large‐scale exercise, it may not fully reflect all complexities present in real disasters. Gaining research access to real incidents is unlikely due to their unpredictability and risks posed to safety. However, the national live exercise was developed by emergency service practitioners in order to be immersive and to parallel the many complexities of real disasters as closely as possible. Given that the focus of this research was on information sharing in disasters, this may also raise questions regarding generalizability to other contexts. However, MTSs are frequently formed to deal with a wide range of challenges in extreme environments such as financial crises and health care issues, in addition to public security and safety. These environments share many similar features such as time pressure, lack of, excessive or incomplete information, risk, and uncertainty, thereby increasing the relevance of findings to wider audiences.

### Practical implications

In recognition of the difficulties of interagency communication during disasters, the UK Home Office introduced the national Joint Emergency Services Interoperability Programme (JESIP) in 2012. To date, JESIP has predominantly focused on Category 1 responders rather than wider support agencies, adopting strategies such as use of joint aide memoires and universal lexicon, in line with the recommendations of knowledge boundaries research (Kotlarsky *et al*., 2015). Findings of this study highlight that it may be beneficial to expand the remit of this training to include Category 2 responders to improve the ability of all agencies that could be involved in disaster response to share information across the wider network.

Findings also suggest that it may be beneficial to introduce training programmes to promote communication behaviours that facilitate common frames for interpreting messages and understanding information needs. These behaviours include providing a rationale for information shared and requested, along with clarifying roles and remits, but future research may identify additional concrete behaviours that facilitate the development of these common frames. The value of this approach is that training can be delivered within agencies, and more frequently than large‐scale exercises, but with benefits for both intra‐ and interagency communication. Previous research into developing common frames‐of‐reference for use of language in military contexts demonstrates that even short periods of classroom‐based training (less than an hour) can increase information sharing and coordination (Firth *et al*., 2015). However, it is also important to recognize the practical limitations for adopting these types of communication behaviours. For example, the time pressures associated with decision‐making in extreme environments may sometimes make it difficult to provide rationales for why information is needed or shared. Further research should examine the impact of adopting such practices on cognitive load within the context of complex and time‐critical environments.

Another potential avenue for supporting information sharing may be to utilize technology, including instant messaging functions. These systems allow users to exchange time‐stamped text, audio, picture and video messages securely and instantly across geographical locations, enabling information to be shared across Command levels regardless of whether meetings overlap. Sharing images and videos may also enable Tactical and Strategic levels to gain a more informative overview of the incident ground during the early phases without physically attending the incident. However, protocols would still need to be established to develop shared frames for promoting the effective exchange of information across this system, similar to those discussed above. It may also be necessary to assign dedicated roles (similar to loggists who are responsible for keeping records of decision processes) as it would be cognitively demanding to both monitor and update systems in addition to engaging in discussions during meetings.

The corporate domain has adopted a range of other technological support tools to promote information sharing, from providing remote team members with the ability to determine when to contact other members, to the management of shared activity (Scott, Cummings, Graeber, Nelson, & Bolia, 2006). Technological systems could be adapted to incorporate the nuances required at each command level of disaster response. For example, at an operational level exact geolocation information could be incorporated into remote team member tracking and imposed onto a map of the incident area. This would provide Tactical and Strategic levels with an understanding of where their personnel currently are, what their current task is and if they are available to provide an update without interrupting their task. Both medical and military domains are already making use of technologies in similar ways (Bardram & Bossen, 2005; Fitzpatrick & Ellingsen, 2012), and it would be beneficial for future disaster response and communication research to draw on these domains to identify where adaptive technology could support DSA in *ad hoc* MTS.

## References

[joop12217-bib-0001] Alison, L. , Power, N. , van den Heuvel, C. , Humann, M. , Palasinksi, M. , & Crego, J. (2015). Decision inertia: Deciding between least‐worst outcomes in emergency responses to disasters. Journal of Occupational & Organizational Psychology, 88, 295–321. 10.1111/joop.12108

[joop12217-bib-0002] Alison, L. , van den Heuvel, C. , Waring, S. , Power, N. , Long, A. , O'Hara, T. , & Crego, J. (2013). Immersive simulated learning environments for researching critical incidents. A knowledge synthesis of the literature and experiences of studying high‐risk strategic decision making. Journal of Cognitive Engineering & Decision Making, 7, 255–272. 10.1177/1555343412468113

[joop12217-bib-0003] Allison, B. B. , & Shuffler, M. L. (2014). Getting the “I” out of multiteam systems: A case study from the financial services industry In Shuffler M. L., Salas E. & Rico R. (Eds.), Pushing the boundaries: Multiteam systems in research and practice (pp. 187–206). Bingley, UK: Emerald.

[joop12217-bib-0004] Baber, C. , Attfield, S. , Conway, G. , Rooney, C. , & Kodagoda, N. (2016). Collaborative sense‐making during simulated Intelligence Analysis Exercises. International Journal of Human‐Computer Studies, 86, 94–108. 10.1016/j.ijhcs.2015.10.001

[joop12217-bib-0005] Bardram, J. E. , & Bossen, C. (2005). Mobility work: The spatial dimension of collaboration at a hospital. Computer Supported Cooperative Work, 14, 131–160. 10.1007/s10606-005-0989-y

[joop12217-bib-0006] Bashir, M. , Afzal, M. T. , & Azeem, M. (2008). Reliability and validity of qualitative and operational research paradigm. Pakistan Journal of Statistics & Operation Research, 4(1), 35–45. 10.18187/pjsor.v4i1.59

[joop12217-bib-0007] Bechky, B. A. (2003). Sharing meaning across occupational communities: The transformation of understanding on a production floor. Organizational Science, 14, 312–330. 10.1287/orsc.14.3.312.15162

[joop12217-bib-0008] Berggren, P. , Johansson, B. J. E. , Baroutsi, N. , Turcotte, I. , & Tremblay, S. (2014). Assessing team focused behaviors in emergency response teams using the shared priorities measure. Proceedings of the 11th ISCRAM. The Pennsylvania State University, University Park, PA.

[joop12217-bib-0009] Boland, R. J. , & Tenkasi, R. V. (1995). Perspective making and perspective taking in communities of knowing. Organizational Science, 6, 350–372. 10.1287/orsc.6.4.350

[joop12217-bib-0010] Braun, V. , & Clarke, V. (2006). Using thematic analysis in psychology. Qualitative Research in Psychology, 3, 77–101. 10.1191/1478088706qp063oa

[joop12217-bib-0011] Burke, C. S. , Salas, E. , Estep, S. , & Pierce, L. (2007). Facilitating team adaptation “in the wild”: A theoretical framework, instructional strategies, and research agenda In Hoffman R. (Ed.), Expertise out of context (pp. 507–524). New York, NY: Taylor & Francis Group.

[joop12217-bib-0012] Cooke, N. J. , Kiekel, P. A. , Salas, E. , Stout, R. , Bowers, C. , & Cannon‐Bowers, J. (2003). Measuring team knowledge: A window to the cognitive underpinnings of team performance. Journal of Applied Psychology, 7, 179–199. 10.1037/1089-2699.7.3.179

[joop12217-bib-0013] Cronin, M. A. , Bezrukova, K. , Weingart, L. R. , & Tinsley, C. H. (2011). Subgroups within a team: The role of cognitive and affective integration. Journal of Organizational Behavior, 32, 831–849. 10.1002/job.707

[joop12217-bib-0014] Cronin, M. A. , & Weingart, L. R. (2007). Representational gaps, information processing, and conflict in functionally diverse teams. Academy of Management Review, 32, 761–773. 10.5465/AMR.2007.25275511

[joop12217-bib-0015] Cuijpers, M. , Uitdewilligen, S. , & Guenter, H. (2016). Effects of dual identification and interteam conflict on multiteam system performance. Journal of Occupational & Organizational Psychology, 89(1), 141–171. 10.1111/joop.12113

[joop12217-bib-0016] Davison, R. B. , Hollenbeck, J. R. , Barnes, C. M. , Sleesman, D. J. , & Ilgen, D. R. (2012). Coordinated action in multiteam systems. Journal of Applied Psychology, 97, 808–824. 10.1037/a0026682 22201246

[joop12217-bib-0017] de Vries, T. , Walter, F. , van der Vegt, G. , & Essens, P. (2014). Antecedents of individuals’ interteam coordination: Broad functional experiences as a mixed blessing. Academy of Management Journal, 57, 1334–1359. 10.5465/amj.2012.0360

[joop12217-bib-0018] DeChurch, L. A. , Burke, C. S. , Shuffler, M. L. , Lyons, R. , Doty, D. , & Salas, E. (2011). A historiometric analysis of leadership in mission critical multiteam environments. The Leadership Quarterly, 22, 152–169. 10.1016/j.leaqua.2010.12.013

[joop12217-bib-0019] DeCostanza, A. , DiRosa, G. , Jiménez‐Rodríguez, M. , & Cianciolo, A. (2014). No mission too difficult: Army units within exponentially complex multiteam systems In Shuffler M. L., Salas E. & Rico R. (Eds.), Pushing the boundaries: Multiteam systems in research and practice (pp. 61–76). Bingley, UK: Emerald 10.1108/rmgt

[joop12217-bib-0020] Dierdorff, E. D. , Surface, E. A. , & Brown, K. G. (2010). Frame‐of‐reference training effectiveness: Effects of goal orientation and self‐efficacy on affective, cognitive, skill‐based, and transfer outcomes. Journal of Applied Psychology, 95, 1181–1191. 10.1037/a0020856 20853944

[joop12217-bib-0021] Endsley, M. R. (1995). Toward a theory of situation awareness in dynamic systems. Human Factors, 37(1), 32–64. 10.1518/001872095779049543

[joop12217-bib-0022] Endsley, M. R. (2000). Theoretical underpinnings of situation awareness: A critical review, in situation awareness analysis and measurement In Endsley M. R. & Garland F. J. (Eds.), Situation awareness analysis and measurement (pp. 3–32). Mahwah, NJ: Lawrence Erlbaum Associates.

[joop12217-bib-0023] Endsley, M. R. (2015). Situation awareness. Journal of Cognitive Engineering & Decision Making, 9(1), 4–32. 10.1177/1555343415572631

[joop12217-bib-0024] Endsley, M. R. , & Jones, W. M. (1997). Situation awareness, information dominance, and information warfare (No. AL/CF‐TR‐1997‐0156). Wright‐Patterson Air Force Base, OH: United States Air Force Armstrong Laboratory.

[joop12217-bib-0025] Firth, B. M. , Hollenbeck, J. R. , Miles, J. E. , Ilgen, D. R. , & Barnes, C. M. (2015). Same page, different books: Extending representational gaps theory to enhance performance in multiteam systems. Academy of Management Journal, 58, 813–835. 10.5465/amj.2013.0216

[joop12217-bib-0026] Fitzpatrick, G. , & Ellingsen, G. (2012). A review of 25 years of CSCW research in healthcare: Contributions, challenges and future agendas. Computer Supported Cooperative Work, 22, 609–665. 10.1007/s10606-012-9168-0

[joop12217-bib-0027] Frith, H. , & Gleeson, K. (2004). Clothing and embodiment: Men managing body image and appearance. Psychology of Men & Masculinity, 5(1), 40–48. 10.1037/1524-9220.5.1.40

[joop12217-bib-0028] Gelo, O. , Braakmann, D. , & Benetka, G. (2008). Quantitative and qualitative research: Beyond the debate. Integrative Psychological & Behavioral Science, 42, 266–290. 10.1007/s12124-008-9078-3 18795385

[joop12217-bib-0029] Goodwin, G. F. , Essens, P. J. M. D. , & Smith, D. (2012). Multiteam systems in the public sector In Zaccaro S. J., Marks M. A. & DeChurch L. (Eds.), Multiteam systems: An organization form for dynamic and complex environments (pp. 53–80). New York, NY: Routledge Taylor & Francis Group.

[joop12217-bib-0030] Gore, J. , Flin, R. , Stanton, N. , & Wong, B. L. W. (2015). Applications for naturalistic decision‐making. Journal of Occupational & Organizational Psychology, 88, 223–230. 10.1111/joop.12121 PMC6020850 29962664

[joop12217-bib-0031] Heavey, C. , & Simsek, Z. (2015). Transactive memory systems and firm performance: An upper echelons perspective. Organization Science, 26, 941–959. 10.1287/orsc.2015.0979

[joop12217-bib-0032] Hollenbeck, J. R. , Beersma, B. , & Schouten, M. E. (2012). Beyond team types and taxonomies: A dimensional scaling conceptualization for team description. Academy of Management Review, 37(1), 82–106. 10.5465/amr.2010.0181

[joop12217-bib-0033] House, A. , Power, N. , & Alison, L. (2014). A systematic review of the potential hurdles of interoperability to the emergency services in major incidents: Recommendations for solutions and alternatives. Cognition, Technology & Work, 16, 319–335. 10.1007/s10111-013-0259-6

[joop12217-bib-0034] Hsieh, H. , & Shannon, S. E. (2005). Three approaches to qualitative content analysis. Qualitative Health Research, 15, 1277–1288. 10.1177/1049732305276687 16204405

[joop12217-bib-0035] Jarvenpaa, S. L. , & Keating, E. (2011). Hallowed grounds: The role of cultural values, practices, and institutions in TMS in an offshored complex engineering services project. IEEE Transactions on Engineering Management, 58, 786–798. 10.1109/TEM.2010.2091133

[joop12217-bib-0036] Jones, M. V. , Coviello, N. , & Tang, Y. K. (2011). International entrepreneurship research (1989–2009): A domain ontology and thematic analysis. Journal of Business Venturing, 26, 632–659. 10.1016/j.jbusvent.2011.04.001

[joop12217-bib-0037] Klein, G. (2008). Naturalistic decision making. Human Factors, 50, 456–460. 10.1518/001872008X288385 18689053

[joop12217-bib-0038] Klein, G. , Moon, B. M. , & Hoffman, R. R. (2006a). Making sense of sense‐making 1: Alternative perspectives. IEEE Intelligent Systems, 21, 70–73. 10.1109/MIS.2006.75

[joop12217-bib-0039] Klein, G. , Moon, B. M. , & Hoffman, R. R. (2006b). Making sense of sense‐making 2: A macro‐cognitive model. IEEE Intelligent Systems, 21, 88–92. 10.1109/MIS.2006.100

[joop12217-bib-0040] Klein, K. J. , Ziegert, J. C. , Knight, A. P. , & Xiao, Y. (2006). Dynamic delegation: Shared hierarchical and deindividualized leadership in extreme action teams. Administrative Science Quarterly, 51, 590–621. 10.2189/asqu.51.4.590

[joop12217-bib-0041] Kotlarsky, J. , van den Hooff, B. , & Houtman, L. (2015). Are we on the same page? Knowledge boundaries and transactive memory system development in cross‐functional teams. Communication Research, 42, 319–344. 10.1177/0093650212469402

[joop12217-bib-0042] Kozlowski, S. W. J. , & Ilgen, D. R. (2006). Enhancing the effectiveness of work groups and teams. Psychological Science in the Public Interest, 7(3), 77–124. 10.1111/j.1529-1006.2006.00030.x 26158912

[joop12217-bib-0043] LePine, J. A. , Piccolo, R. F. , Jackson, C. L. , Mathieu, J. E. , & Saul, J. R. (2008). A meta‐analysis of teamwork processes: Tests of a multidimensional model and relationships with team effectiveness criteria. Personnel Psychology, 61, 273–307. 10.1111/j.1744-6570.2008.00114.x

[joop12217-bib-0044] Lipshitz, R. , Klein, G. , Orasanu, J. , & Salas, E. (2001). Focus article: Taking stock of naturalistic decision making. Journal of Behavioral Decision Making, 14, 331–352. 10.1002/(ISSN)1099-0771

[joop12217-bib-0045] London Emergency Services Liaison Panel . (2015). Major incident procedure manual (9.3 ed.). Retrieved from https://www.met.police.uk/globalassets/foi-media/priorities_and_how_we_are_doing/corporate/specialist-crime-operations-leslp-major-incident-procedure-manual

[joop12217-bib-0046] Luvison, D. , & Marks, M. A. (2012). Team coordination in strategic alliances: Identifying conditions that reduce team willingness to cooperate. International Journal of Strategic Business Alliances, 3(1), 1–22. 10.1504/IJSBA.2013.058290

[joop12217-bib-0047] Marks, M. A. , DeChurch, L. A. , Mathieu, J. E. , Panzer, F. J. , & Alonso, A. (2005). Teamwork in multiteam systems. Journal of Applied Psychology, 90, 964–971. 10.1037/0021-9010.90.5.964 16162068

[joop12217-bib-0048] Martin, A. , & Bal, V. (2006). The state of teams. Greensboro, NC: Center for Creative Leadership.

[joop12217-bib-0049] Mathieu, J. E. , Marks, M. A. , & Zaccaro, S. J. (2001). Multiteam systems In Anderson N., Ones D. S., Sinangil H. K. & Viswesvaran C. (Eds.), Handbook of industrial, work and organizational psychology, vol. 2: Organizational psychology (pp. 289–313). London, UK: Sage Publications.

[joop12217-bib-0050] Maynard, M. T. , Mathieu, J. , Rapp, T. L. , & Gilson, L. L. (2012). Something(s) old and something(s) new: Modeling drivers of global virtual team effectiveness. Journal of Organizational Behavior, 33, 342–365. 10.1002/job.1772

[joop12217-bib-0051] McGraw, K. O. , & Wong, S. P. (1996). Forming inferences about some interclass correlation coefficients. Psychological Methods, 1(1), 30–46. 10.1037/1082-989X.1.1.30

[joop12217-bib-0052] McLeod, J. (2001). Qualitative research in counselling and psychotherapy. London, UK: Sage Publishing 10.4135/9781849209663

[joop12217-bib-0053] Mendonça, D. , Jefferson, T. , & Harrald, J. (2007). Emergent Interoperability: Collaborative adhocracies and mix and match technologies in emergency management. Communications of the ACM – Emergency Response Information Systems: Emerging Trends and Technologies, 50(3), 44–49. 10.1145/1226736.1226764

[joop12217-bib-0054] Nazir, S. , Sorensen, L. J. , Overgård, K. I. , & Manca, D. (2014). How distributed situation awareness influences process safety. Chemical Engineering Transactions, 36, 409–414. 10.3303/CET1436069

[joop12217-bib-0055] Pollock, K. (2013). Review of persistent lessons identified relating to interoperability from emergencies and major incidents since 1986. Retrieved from http://www.jesip.org.uk/uploads/media/pdf/Pollock_Review_Oct_2013.pdf

[joop12217-bib-0056] Power, N. , & Alison, L. (2016). Offence or defence? Approach and avoid goals in the multi‐agency emergency response to simulated terrorism attack. Journal of Occupational & Organizational Psychology, 90(1), 51–76. 10.1111/joop.12159

[joop12217-bib-0057] Rankin, A. , Dahlbäck, N. , & Lundbery, J. (2013). A case study of factor influencing role improvisation in crisis response teams. Cognition, Technology & Work, 15, 79–93. 10.1007/s10111-011-0186-3

[joop12217-bib-0058] Ren, Y. , & Argote, L. (2011). Transactive memory systems 1985–2010: An integrative framework of key dimensions, antecedents, and consequences. Academy of Management Annals, 5, 189–229. 10.1080/19416520.2011.590300

[joop12217-bib-0059] Rentsch, J. R. , & Staniewicz, M. J. (2012). Cognitive similarity configurations in multiteam systems In Zaccaro S. J., Marks M. A. & DeChurch L. A. (Eds.), Multiteam systems: An organizational form for dynamic and complex environments (pp. 225–253). New York, NY: Routledge.

[joop12217-bib-0060] Salmon, P. M. , Stanton, N. A. , Walker, G. H. , & Jenkins, D. P. (2009). Distributed situation awareness: Theory, measurement and application to teamwork. Aldershot, UK: Ashgate.

[joop12217-bib-0061] Sandhåland, H. , Oltedal, H. A. , Hystad, S. W. , & Eid, J. (2015). Distributed situation awareness in complex collaborative systems: A field study of bridge operations on platform supply vessels. Journal of Occupational & Organizational Psychology, 88, 273–294. 10.1111/joop.12111 26028823 PMC4440387

[joop12217-bib-0062] Saner, L. D. , Bolstad, C. A. , Gonzalez, C. , & Cuevas, H. M. (2009). Measuring and predicting shared situation awareness in teams. Journal of Cognitive Engineering & Decision Making, 3, 280–308. 10.1518/155534309X474497

[joop12217-bib-0063] Schleicher, D. J. , & Day, D. V. (1998). A cognitive evaluation of frame‐of‐reference training: Content and process issues. Organizational Behavior & Human Decision Processes, 73, 76–101. 10.1006/obhd.1998.2751 9705795

[joop12217-bib-0064] Schmidt, A. , & DeShon, R. P. (2007). What to do? The effects of discrepancies, incentives, and time on dynamic goal prioritization. Journal of Applied Psychology, 92, 928–941. 10.1037/0021-9010.92.4.928 17638455

[joop12217-bib-0065] Scholtens, A. (2008). Controlled collaboration in disaster and crisis management in the Netherlands, history and practice of an overestimated and underestimated concept. Journal of Contingencies & Crisis Management, 16, 195–207. 10.1111/j.1468-5973.2008.00550.x

[joop12217-bib-0066] Schraagen, J. M. C. , Huis in ‘t Veld, M. , & De Koning, L. (2010). Information sharing during crisis management in hierarchical vs. network teams. Journal of Contingencies & Crisis Management, 18(2), 117–127. 10.1111/j.1468-5973.2010.00604.x

[joop12217-bib-0067] Schraagen, J. M. C. , & Van de Ven, J. G. M. (2011). Human factors aspects of ICT for crisis management. Cognition, Technology & Work, 13, 175–187. 10.1007/s10111-011-0175-6

[joop12217-bib-0068] Scott, S. D. , Cummings, M. L. , Graeber, D. S. , Nelson, W. T. , & Bolia, R. S. (2006). Collaboration technology in military team operations: lessons learned from the corporate domain. Proceedings of CCRTS 2006: the command and control research and technology symposium, San Diego, CA.

[joop12217-bib-0069] Shuffler, M. L. , Jiménez‐Rodríguez, M. , & Kramer, W. (2015). The science of multiteam systems: A review and future research agenda. Small Group Research, 46, 659–699. 10.1177/1046496415603455

[joop12217-bib-0070] Stanton, N. A. (2016). Distributed situation awareness. Theoretical Issues in Ergonomics Science, 17(1), 1–7. 10.1080/1463922X.2015.1106615

[joop12217-bib-0071] Stanton, N. A. , Stewart, R. , Harris, D. J. , Houghton, R. J. , Baber, C. , McMaster, R. , … Green, D. (2006). Distributed situation awareness in dynamic systems: Theoretical development and application of an ergonomics methodology. Ergonomics, 49, 1288–1311. 10.1080/00140130600612762 17008257

[joop12217-bib-0072] The Civil Contingencies Act. (2004). Retrieved from https://www.legislation.gov.uk/ukpga/2004/36/contents

[joop12217-bib-0073] Wegner, D. M. (1987). Transactive memory: A contemporary analysis of the group mind In Mullen B. & Goethals G. R. (Eds.), Theories of group behavior (pp. 185–208). New York, NY: Springer‐Verlag 10.1007/978-1-4612-4634-3

[joop12217-bib-0074] Wegner, D. M. , Guiliano, T. , & Hertel, P. (1985). Cognitive interdependence in close relationships In Ickes W. J. (Ed.), Compatible and incompatible relationships (pp. 253–276). New York, NY: Springer 10.1007/978-1-4612-5044-9

[joop12217-bib-0075] Weingart, L. R. , Cronin, M. A. , Houser, C. J. S. , Cagan, J. , & Vogel, C. M. (2005). Functional diversity and conflict in cross‐functional product development teams: Considering representational gap and task characteristics. Research in Management, 4, 89–110.

[joop12217-bib-0076] Wright, M. C. , Taekman, J. M. , & Endsley, M. R. (2004). Objective measures of situation awareness in a simulated medical environment. Quality & Safety in Health Care, 13, 65–71. 10.1136/qshc.2004.009951 PMC1765787 15465958

[joop12217-bib-0077] Zika‐Viktorsson, A. , Sundström, P. , & Engwall, M. (2006). Project overload: An exploratory study of work and management in multi‐project settings. International Journal of Project Management, 24, 385–394. 10.1016/j.ijproman.2006.02.010

